# Survey dataset on the externalizing self-esteem and gender effects on self-esteem subscales of students in Zabol University of Medical Sciences, Iran

**DOI:** 10.1016/j.dib.2018.10.019

**Published:** 2018-10-09

**Authors:** Maryam Ghaljahi, Somayeh Rahdar, Seyedeh Zeynab Almasi, Shahin Ahmadi, Chinenye Adaobi Igwegbe

**Affiliations:** a Department of Occupational Health Engineering, School of Public Health, Zabol University of Medical Science, Zabol, Iran; b Department of Environmental Health Engineering, School of Public Health, Zabol University of Medical Science, Zabol, Iran; c Research Center Health, School of Public Health, Zahedan University of Medical Science, Zahedan, Iran; d Department of Chemical Engineering, Nnamdi Azikiwe University, Awka, Nigeria

**Keywords:** Self-esteem, Gender, Zabol University Students, Data mining, Descriptive statistics

## Abstract

The data presents the self-esteem examination of undergraduate students studying in Zabol University of Medical Sciences, Iran in 2017 and its relationship with gender. The total number of participants was 100 (49% female and 51% male). The 100 students were selected through random sampling method. The average age of participants was 21.61 years while the youngest and the oldest participants were 19 and 32 years old, respectively. The data were collected using the Coopersmith Self-Esteem Inventory (CSEI) and analyzed by descriptive statistics (frequency, mean, standard deviation, minimum and maximum) using SPSS version 22 (statistical package for Social Sciences). The detailed dataset is presented in this paper.

**Specifications table**

**Table:** 

Subject area	Social Sciences
More specific subject area	Quantitative Psychology
Type of data	Table and figure
How data was acquired	Field Survey: The required data were collected through the Coopersmith Self-Esteem Inventory (CSEI) and analyzed via descriptive statistics using statistics using SPSS version 22 (statistical package for Social Sciences).
Data format	Raw and analyzed
Experimental factors	The total number of participants was 100 (49% female and 51% male) undergraduate university students, which was selected randomly.
Experimental features	The descriptive and inferential statistics on the self-esteem examination of university students studying at Zabol University of Medical Sciences, Iran and its relationship with gender.
Data source location	The data was collected from Zabol University of Medical Sciences, Zabol, Iran (Latitude 31.0287°N, Longitude 61.5012°E)
Data accessibility	Data is within this article.

**Value of the data**

• An insight into the relationship between self-esteem and gender will be provided.
• It will encourage relating gender to other psychological research problems.
• The data will also serve as a reference for other researchers in the same field.

## 1 Data

**Fig. 1:**
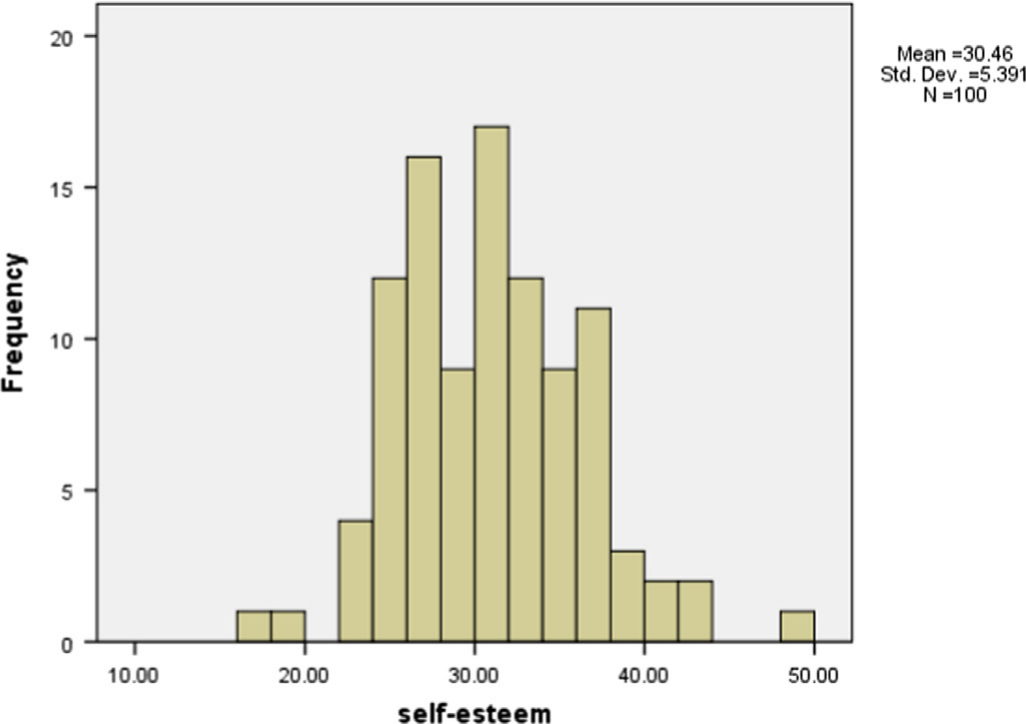
The hypothetical distribution of self-esteem scores on the Coopersmith self-esteem.

**Fig. 2:**
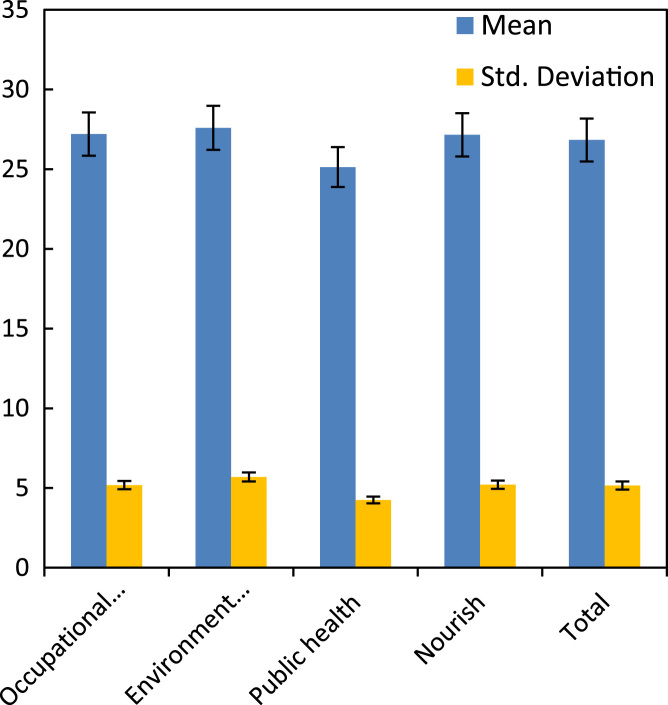
Mean self-esteem and standard deviation by field of study of the participants.

**Fig. 3:**
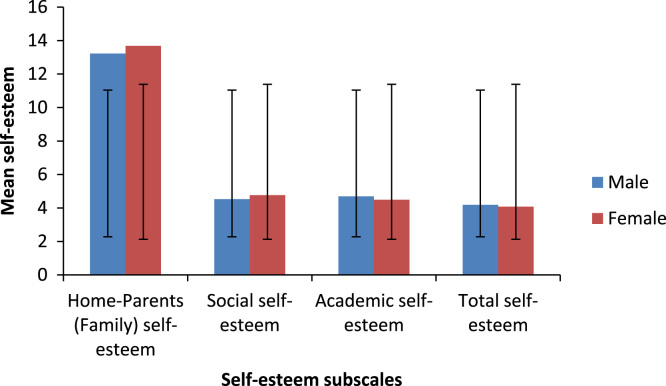
Mean self-esteem based on the self-esteem subscales by gender of the participants.

Many psychologists believe that men and women are fundamentally different and the male and female constructs are entirely distinct [1]. Moreover, self-esteem is related to several factors, and different studies in different societies have shown different results [2]. Self-esteem is defined as an individual׳s overall evaluation of his/her self and his/her level of self-satisfaction. Self-esteem is also a feeling of self-worth, happiness, and capability [3], [4], [5]. Self-esteem generally affects the performance of an individual in all aspects of life, for example, performance in academics, dissipation of duties in a workplace, health and positive thinking. It also affects the social and mental well-being of an individual [6]. The data were collected through the Coopersmith Self-Esteem Inventory (CSEI) which was developed by Coppersmith in 1967 [7]. The data in this article is a set of responses solicited from 100 (51 females and 49 males) students in Zabol University of Medical Sciences, Iran. The details of the sample size are shown in Table 1. The hypothetical distribution of self-esteem scores on the Coopersmith self-esteem is presented in Fig. 1. The descriptive statistics for the gender differences in the distribution of the total self-esteem for the school students showing mean, standard deviation, minimum, maximum, range and total number of samples is shown in Table 2, Table 3, Table 4, Table 5, Table 6, Table 7 and Fig. 2, Fig. 3. The data shows the relationship between gender, age, different educational groups (Occupational health, Environmental health, Public health and Nourish), and self-esteem subscales (General self-esteem, Home-Parents (Family) self-esteem, Social self-esteem, Academic self-esteem, and Home-Parents (Family) self-esteem) of university students.

**Table 1:** Demographic characteristics of the participants (students).

	**Gender**	**Number (*N*)**	**Age**	
			19–23	<23
**Occupational health**	Female	21	21	–
	Male	14	11	3
**Environmental health**	Female	15	15	–
	Male	14	–	14
**Public health**	Female	13	7	6
	Male	10	8	2
**Nourish**	Female	2	1	1
	Male	11	11	–

**Table 2:** Percentage distribution of gender of the participants.

	**Frequency**	**Percent**	**Valid percent**	**Cumulative percent**
**Male**	49	49.0	49.0	49.0
**Female**	51	51.0	51.0	100.0
**Total**	100	100.0	100.0

**Table 3:** Descriptive statistics of the students based on different educational group and self-esteem subscales.

**Study**		***N***	**Minimum**	**Maximum**	**Mean**	**Std. deviation**
**Occupational health**	General self-esteem	35	6.00	19.00	13.7143	2.79255
	Home-Parents (Family) self-esteem	35	1.00	7.00	4.6857	1.36708
	Social self-esteem	35	1.00	7.00	4.7429	1.55947
	Academic self-esteem	35	2.00	7.00	4.0571	1.37076
	Total self-esteem	35	15.00	38.00	27.2000	5.18368
**Environmental health**	General self-esteem	29	8.00	22.00	13.5517	3.45983
	Home-Parents (Family) self-esteem	29	2.00	7.00	4.9655	1.37536
	Social self-esteem	29	1.00	7.00	4.6897	1.83427
	Academic self-esteem	29	1.00	8.00	4.3793	1.63475
	Total self-esteem	29	17.00	42.00	27.5862	5.69158
**Public health**	General self-esteem	23	6.00	19.00	12.6087	3.04122
	Home-Parents (Family) self-esteem	23	0.00	7.00	4.0435	1.46095
	Social self-esteem	23	3.00	7.00	4.4348	1.03687
	Academic self-esteem	23	2.00	12.00	4.0435	2.03332
	Total self-esteem	23	19.00	36.00	25.1304	4.24590
					
**Nourish**	General self-esteem	13	8.00	17.00	14.0769	2.98501
	Home-Parents (Family) self-esteem	13	2.00	6.00	4.9231	1.25576
	Social self-esteem	13	0.00	7.00	4.2308	1.96443
	Academic self-esteem	13	0.00	6.00	3.9231	1.70595
	Total self-esteem	13	19.00	34.00	27.1538	5.20970

**Table 4:** Descriptive statistics of the students based on gender and total self-esteem subscales.

**Gender**		*N*	Minimum	Maximum	Mean	Std. deviation
**Male**	General self-esteem	49	6.00	21.00	13.2245	3.29940
	Home-Parents (Family) self-esteem	49	0.00	7.00	4.5306	1.35558
	Social self-esteem	49	0.00	7.00	4.6939	1.64828
	Academic self-esteem	49	0.00	12.00	4.1837	1.87831
	Total self-esteem	49	15.00	42.00	26.6327	5.27452
**Female**	General self-esteem	51	8.00	22.00	13.6863	2.85300
	Home-Parents (Family) self-esteem	51	1.00	7.00	4.7647	1.45035
	Social self-esteem	51	1.00	7.00	4.4902	1.54107
	Academic self-esteem	51	2.00	7.00	4.0784	1.39776
	Total self-esteem	51	15.00	39.00	27.0196	5.08523

**Table 5:** Descriptive statistics on self-esteem subscales of the students based on age.

	**Age**	***N***	**Mean**	**Std. deviation**	**Std. Error Mean**
**General self-esteem**	19–23	74	13.5405	3.03039	0.35228
	<23	26	13.2308	3.24108	0.63563
**Home-Parents (Family) self-esteem**	19–23	74	4.5270	1.45454	0.16909
	<23	26	5.0000	1.20000	0.23534
**Social self-esteem**	19–23	74	4.6486	1.58272	0.18399
	<23	26	4.4231	1.62906	0.31949
**Academic self-esteem**	19–23	74	4.0946	1.45403	0.16903
	<23	26	4.2308	2.12241	0.41624
**Total self-esteem**	19–23	74	26.8108	5.30359	0.61653
	<23	26	26.8846	4.81104	0.94352

**Table 6:** Descriptive statistics based on self-esteem subscales and gender.

**Group statistics**	**Gender**	***N***	**Mean**	**Std. deviation**	**Std. Error Mean**
General self-esteem	Male	49	13.22 ± 3.29	3.29940	0.47134
	Female	51	13.68 ± 2.85	2.85300	0.39950
Home-Parents (Family) self-esteem	Male	49	4.53 ± 1.35	1.35558	0.19365
	Female	51	4.76 ± 1.45	1.45035	0.20309
Social self-esteem	Male	49	4.69 ± 1.64	1.64828	0.23547
	Female	51	4.49 ± 1.54	1.54107	0.21579
Academic self-esteem	Male	49	4.18 ± 1.87	1.87831	0.26833
	Female	51	4.07 ± 1.39	1.39776	0.19573
Total self-esteem	Male	49	26.63 ± 5.27	5.27452	0.75350
	Female	51	27.01 ± 5.08	5.08523	0.71208

**Table 7:** Descriptive statistics on self-esteem based on the field study of the students.

	***N***	**Mean**	**Std. deviation**	**Std. error**	**Minimum**	**Maximum**
**Occupational health**	35	27.2000	5.18368	0.87620	15.00	38.00
**Environmental health**	29	27.5862	5.69158	1.05690	17.00	42.00
**Public health**	23	25.1304	4.24590	0.88533	19.00	36.00
**Nourish**	13	27.1538	5.20970	1.44491	19.00	34.00
**Total**	100	26.8300	5.15626	0.51563	15.00	42.00

## 2 Experimental design, materials, and methods

### 2.1 Study area description

**Fig. 4:**
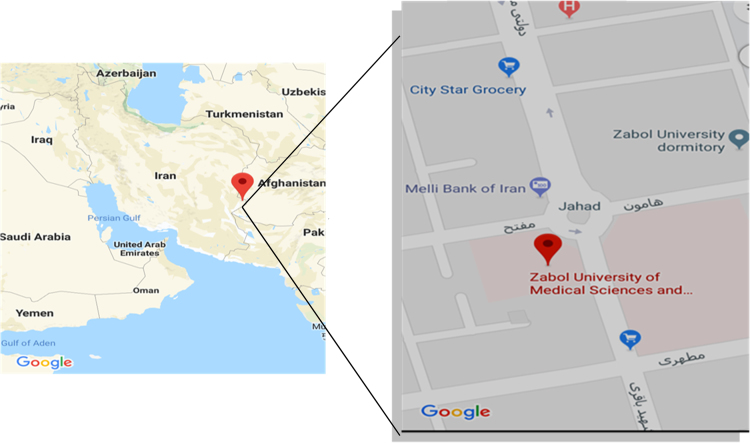
Geospatial map of the region of study.

Zabol city is the capital of Zabol County, Sistan and Baluchestan Province, which lies on the border with Afghanistan, and has a total area of approximately 344 km^2^. The population of Zabol was 137,722 in 2011. Fig. 4 shows the geospatial map of the region of study.

### 2.2 Sample collection and analytical procedures

All undergraduate students in the Zabol University of Medical Sciences, Iran in 2017 were included in the present study. A total number of 100 participants (49% female and 51% male) were selected through random sampling method for the study. The average age of participants was 21.61 years, while the youngest and the oldest participants were 19 and 32 years old, respectively. Coopersmith developed his self-esteem inventory based on his revision of Rogers and Dymond׳s self-esteem scale. The data collection tool was a two-part questionnaire: (1) the demographic section covering the participants’ demographic information, such as age, gender, and the field of study; and (2) the Coopersmith Self-Esteem Inventory (CSEI). The participants were also assured that their information would remain confidential. Then, copies of the questionnaire were distributed among the participants to be completed. The CSEI has 58 items; each scored either 1 or 0, so that, positive answers to items 2, 4, 5, 10, 14, 18, 19, 21, 23, 24, 28, 29, 30, 36, 45, and 57 are scored 1, and negative answers are scored 0 while the rest of the items are scored in reverse. Thus, the possible range of scores is 0–50. High scores in the CSEI indicate a high level of self-esteem. The collected data were analyzed by SPSS version 22 computer software. To describe and analyze the collected data, descriptive statistics (frequency, mean, standard deviation, minimum and maximum) was used.
