# Supplementary material for: Survey dataset on the externalizing self-esteem and gender effects on self-esteem subscales of students in Zabol University of Medical Sciences, Iran
Source: Data Brief. 2018 Oct 9;21:407–13. doi: 10.1016/j.dib.2018.10.019 (PMC6198126; doi:10.1016/j.dib.2018.10.019)
Supplement: Supplementary file 1 — Transparency document [file mmc1.docx]

**Conflicts of Interest**

The authors declare that there are no conflicts of interest regarding the publication of this manuscript.
